# Dr. James Rorie

**Published:** 1911-04-15

**Authors:** 


The death is announced at Dundee of Dr. James Rorie, M.D., L.R.C.S.E., formerly Lecturer on Mental Diseases in University College, Dundee, and for forty-five years Medical Superintendent, Dundee Royal Asylum. Dr. Rorie's brilliant career began in 1859, when he gained the M.D. of Edinburgh (thesis gold medal) and the L.R.C.S., the gold medal being but one of the prizes which he won at this time.

